# Proteomic analysis of the anticancer effect of various extracts of endemicThermopsis turcica in human cervical cancer cells

**DOI:** 10.3906/sag-2005-321

**Published:** 2020-12-17

**Authors:** Mustafa YILDIZ, Hakan TERZİ, Saliha Handan YILDIZ, Nuray VAROL, Müjgan ÖZDEMİR ERDOĞAN, Murat KASAP, Nermin AKÇALI, Mustafa SOLAK

**Affiliations:** 1 Department of Molecular Biology and Genetics, Faculty of Science and Literature, Afyon Kocatepe University, Afyonkarahisar Turkey; 2 Department of Medical Genetics, Faculty of Medicine, Afyonkarahisar University of Health Sciences, Afyonkarahisar Turkey; 3 Department of Medical Biology, Faculty of Medicine, Kocaeli University, Kocaeli Turkey

**Keywords:** Anticancer activity, apoptosis, human cervical cancer, proteomics, *Thermopsis turcica*

## Abstract

**Background/aim:**

*Thermopsis* *turcica* is a perennial species endemic to Turkey and different extracts of *T. turcica* have an antiproliferative effect on cancer cells, but there has not been any report on HeLa (human cervical cancer) cells.

**Materials and methods:**

To get a better understanding of the molecular mechanism of anticancer activity of methanolic extracts of leaves (LE) and flowers (FE) of *T. turcica* , we employed 2-DE-based proteomics to explore the proteins involved in anticancer activity in HeLa cells.

**Results:**

*T. turcica* extracts showed a potent cytotoxic effect on HeLa cells with the IC50 values of 1.75 mg/mL for LE and 3.25 mg/mL for FE. The induction of apoptosis by LE and FE was also consistent with increased expression of caspase mRNAs and DNA fragmentation. In terms of the proteomic approach, 27 differentially expressed proteins were detected and identified through MALDI-TOF/TOF mass spectrometry. These altered proteins were involved in cytoskeleton organization and movement, protein folding, proteolysis and translation, cell cycle and proliferation, signal transduction, cell redox homeostasis, and metabolism.

**Conclusion:**

Up-regulation of protein disulfide isomerases and down-regulation of Rho GDP-dissociation inhibitor, heterogeneous nuclear ribonucleoproteins, and heat shock proteins may contribute to the induction of apoptosis and arresting of the cell cycle in HeLa cells.

## 1. Introduction

Cervical cancer has been ranked as the fourth-most common type of cancer in women worldwide; 528,000 new cancer cases and 266,000 deaths are reported annually [1]. It is well-established that almost all cases of cervical cancer result from infections with carcinogenic human papillomavirus. Although prophylactic vaccines were developed for the prevention of this type of cancer, different methods including surgery, chemotherapy, and radiotherapy are used for cancer treatment in developing countries [2]. However, chemotherapeutic treatment in cervical cancer can lead to drug resistance and nonspecific cytotoxicity [3]. Considering that metastatic cervical cancer is incurable, effective anticancer agents are needed.

Plants have been used for thousands of years because they have a large number of secondary metabolites with pharmacological and anticancer properties [4]. Due to their bioactivity, plant secondary metabolites may have a potential action in cancer therapy [5]. Anticancer drugs from plant-derived compounds with pharmacological activities may also improve the efficiency of chemotherapeutic treatments [6]. The *Thermopsis* ( *Fabaceae* ) genus has some species with proven biological activity. For example, various extracts of *T. lanceolata* have potentially toxic effects on mice [7]. Ethanolic extract of *T. rhombifolia* has been shown to have antitumor activity against colon and brain cancers [8]. *Thermopsis* *turcica* , also known as *Vuralia* *turcica* , is an endemic species in Turkey and is considered a medicinal plant. Different extracts of *T. turcica* have been shown to have biological activities such as free radical scavenging, antiinflammatory, mutagenic, and antimicrobial activity [9–11]. Laboratory studies on *T. turcica* indicate the antitumor activity against liver cancer, acute promyelocytic leukemia, and prostate cancer [11,12].

Proteomics is a powerful tool in the investigation of several biological processes including cancer biology. The anticancer mechanisms of plant-derived compounds have been investigated by performing comparative proteome analysis on various types of cancer [13–15]. Xu et al. [13] reported that betulinic acid triggers rapid apoptosis in HeLa cells by invoking ER stress and the mitochondrial-mediated pathway. In a recent study, Khazaei et al. [16] showed that flower extract of *Allium* *atroviolaceum* had cytotoxicity and induce caspase-dependent apoptosis in HeLa cells. However, there has been no report on *T. turcica* extracts affecting human cervical cancer cells, and more research is needed to understand the molecular mechanisms of apoptotic effect. The main purpose of this research was to investigate the potential anticancer effects of the methanolic extracts of *T. turcica* on the human cervical cancer cell line (HeLa) by evaluating cytotoxicity, caspase activity, and apoptosis-related proteins. In this study, a gel-based proteomics approach was used to characterize the proteins involved in apoptosis of HeLa cells treated with *T. turcica* extracts.

## 2. Materials and methods

### 2.1. Plant sample and extract preparation

*Thermopsis* *turcica* plants were collected from the southern coast of Lake Eber of Turkey in May. Flowers and leaves were separated, shade-dried at laboratory conditions, and thoroughly ground. The extraction of samples was performed by an accelerated solvent extraction system (ASE 300, Dionex, USA) with a 100 mL stainless steel cell. The powdered materials were placed in the stainless steel cell and extracted with 70% methanol. Methanolic extracts of flowers (FE) and leaves (LE) were evaporated to remove the solvent in a rotary evaporator and lyophilized in a freeze dryer (Labconco, USA). The dry extracts were solubilized in dimethyl sulfoxide (DMSO, 1%) to obtain a stock solution.

### 2.2. Cell culture and cytotoxicity assay

The HeLa cells were cultured in Dulbecco’s modified Eagle’s medium containing 10% fetal bovine serum. Cells were incubated in 5% CO2 at 37 °C. The cytotoxicity of *T. turcica* on HeLa cells was determined using the WST-1 assay (Roche, Germany). The cervical carcinoma cells (1 × 105/well) were subjected to different concentrations of *T. turcica* extracts or DMSO in 96 well plates for 24 h. At the end of the incubation at 37 °C in 5% CO2, WST-1 solution (10 µL) was added into each well. Optical absorbance was measured at 450 nm with a microplate spectrophotometer (Multiskan GO, Thermo Fisher Scientific, Germany) after incubation at 37 °C for 4 h.

### 2.3. Expression levels of caspase genes

HeLa cells (1 × 106/well) were placed within 6 well plates and subjected to a medium containing IC50 concentrations of FE (3.25 mg/L) and LE (1.75 mg/L) for 24 h. After incubation, the isolation of total RNA was performed using a TriPure isolation reagent (Roche, USA). cDNA was synthesized with an RT2 HT First Strand cDNA synthesis kit (Qiagen, Germany). An RT2 SYBR Green qPCR Green I Master Kit (Qiagen) was used for quantitative analysis of gene expression. The *GAPDH* gene was used to normalize the expression level of apoptosis-related genes *CASP3* , *CASP8* , and *CASP9* . The primers used were 5ʹ -TGGAATTGATGCGTGATGTT-3ʹ and 5ʹ - TGGCTCAGAAGCACACAAAC-3ʹ for *CASP3* , 5ʹ -TCCAAATGCAAACTGGATGA-3ʹ and 5ʹ -TCCCAGGA TGACC CTCTTCT-3ʹ for *CASP8* , 5ʹ -CCA

TATGATCGA GGACAT CCA-3ʹ and 5ʹ -GACTCCCTC

GAGTCTCCA GAT-3ʹ for *CASP9* , and 5ʹ -AGCCACATC

GCTCAGACAC-3ʹ and 5ʹ -GCCCAATACGACCAAATC

C-3ʹ for *GAPDH* .

### 2.4. DNA fragmentation

DNA fragmentation was determined in HeLa cells exposed to *T. turcica* extracts for 24 h. An apoptotic DNA Ladder Kit (Roche Diagnostics International AG, Rotkreuz, ZG, Switzerland) was used for the isolation of fragmented DNA. The DNA fragments were separated on a 2% agarose gel by electrophoresis.

### 2.5. Protein preparation and 2-D gel electrophoresis

HeLa cells (2 × 106) treated with *T. turcica* extracts were harvested by trypsinization and washed three times with cold PBS. The cells were solubilized in 500 µL of a lysis buffer (7 M urea, 2 M thiourea, 60 mM DTT, 4% CHAPS, 1% PMSF, and 0.4% IPG buffer) for 2 h at 4 °C. The suspensions were centrifuged at 16,000 × g for 30 min. After centrifugation, supernatants were collected and stored at -80 °C. Proteins in supernatants were quantified by Bradford assay [17]. In the first dimension of 2-D gel electrophoresis, 50 µg of cell protein was diluted in 300 µL rehydration buffer (lysis buffer with 0.001% bromophenol blue) and was loaded onto IPG strips (17 cm, pH 4–7). Proteins were focused in a Protean IEF system (Bio-Rad) at 80 kVh. Two-dimensional SDS–PAGE was performed in 12% gel on the Protean II XL system (Bio-Rad Laboratories, Inc., Philadelphia, PA, USA). Gels were run under constant amperage (24 mA/gel). After electrophoresis, silver staining was used to visualize proteins in analytical gels [18], whereas protein spots in preparative gels were stained according to Candiano et al. [19].

### 2.6. Image analysis and mass spectrometry

Protein spots were detected and quantified using PDQuest software (Philadelphia, PA, USA). Spots showing significantly and consistently different intensities (more than 1.5-fold and Student’s t-test, P < 0.05) were excised manually from 2-DE gels. The peptide fragments were obtained using an in-gel digestion kit (Thermo Fisher Scientific, Massachusetts, United States). The peptide extracts were concentrated using a ZipTip (Millipore) and eluted in 1 µL of matrix solution (α-cyano-hydroxycinnamic acid). The analyte was spotted onto a MALDI plate and analyzed by AB Sciex TOF/TOF 5800 MS (Applied Biosystems, USA). MS/MS data were submitted to MASCOT for protein identification. The search parameters were as follows: Swiss-Prot; taxonomy, human; trypsin as the digestion enzyme; one missing cleavage; 50 ppm for mass accuracy; and ±0.4 Da for MS/MS tolerance. Carbamidomethylation for cysteine and oxidation for methionine was also considered. A 95% or higher confidence interval was accepted as criteria for protein identifications. Program STRING 9.1 was employed to generate a protein interaction network [20].

### 2.7. Statistical analysis

WST-1 and gene expression analyses data were expressed as mean ± SD. The results were analyzed by the SPSS statistical software package using an analysis of variance (ANOVA). To determine significant differences among means at the significance level of P < 0.05, Duncan’s multiple range test was used.

## 3. Results

### 3.1. Cytotoxic effects of T. turcica extracts in HeLa cells

**Figure 1:**
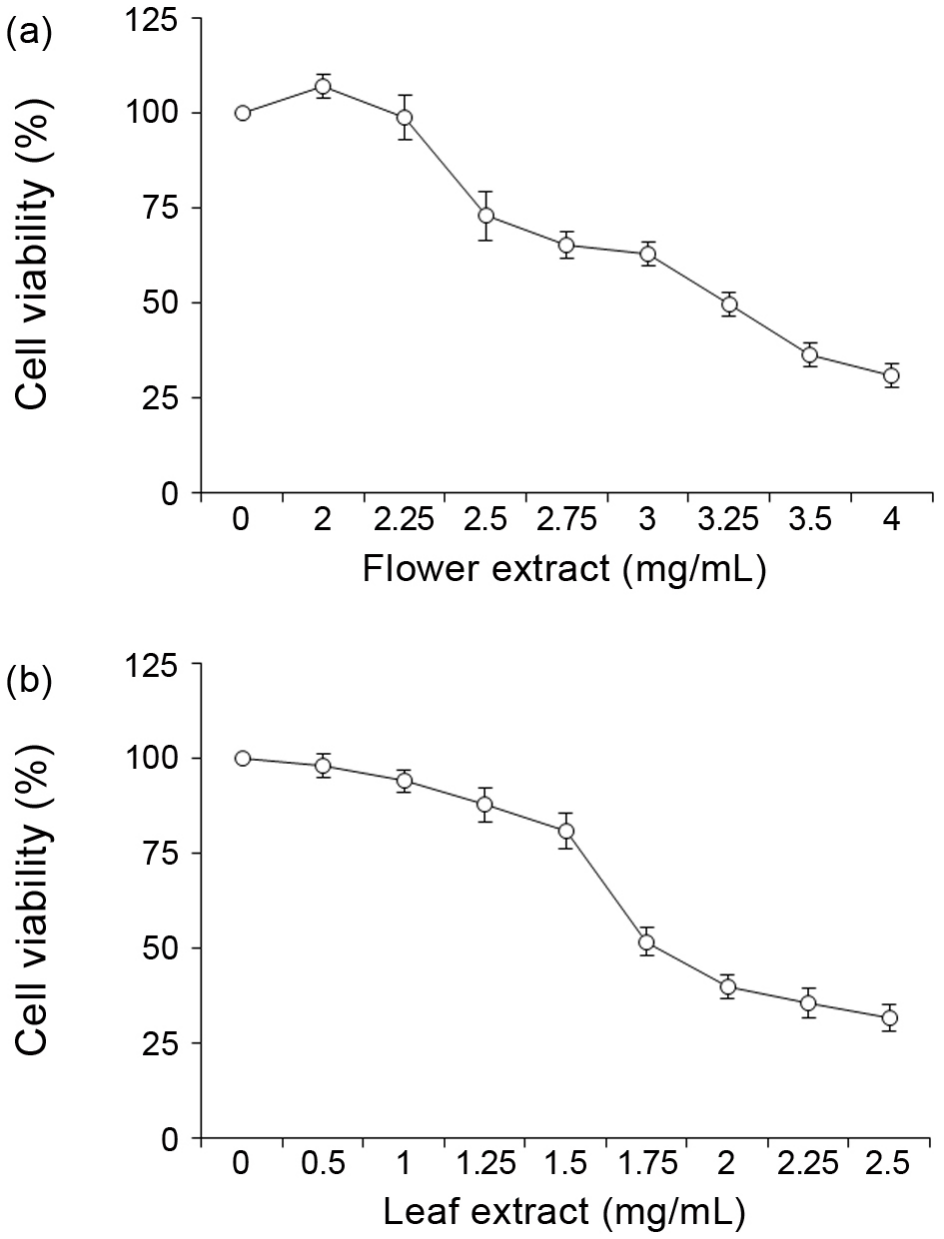
Effects of the flower (a) and leaf (b) extracts of T. turcica on the viability of HeLa cells.

The antiproliferative effects of flower and leaf extracts of *T. turcica* against HeLa cells are shown in Figure 1. We observed that *T. turcica* extracts induced a significant decrease in the viability of HeLa cells with increasing concentration. The IC50 value of FE in HeLa cells was determined as 3.25 mg/mL while the IC50 value of LE was determined as 1.75 mg/mL.

### 3.2. T. turcica extracts induce DNA fragmentation and expression of caspase genes

**Figure 2:**
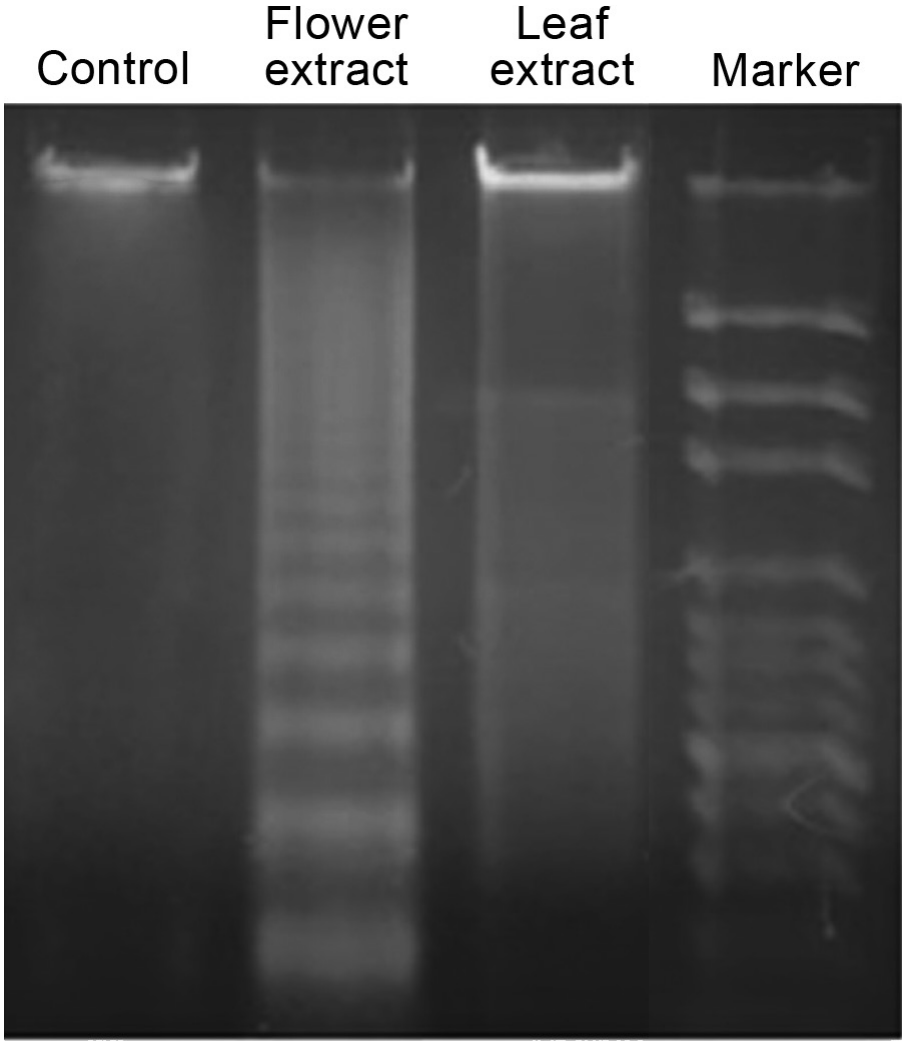
DNA fragmentation of HeLa cells exposed to flower and leaf extracts of T. turcica.

HeLa cells exposed to IC50 of FE and LE for 24 h exhibited DNA laddering patterns in the agarose gels, indicating apoptotic DNA fragmentation (Figure 2). In contrast, no DNA ladder was observed in DMSO treated control.

**Figure 3:**
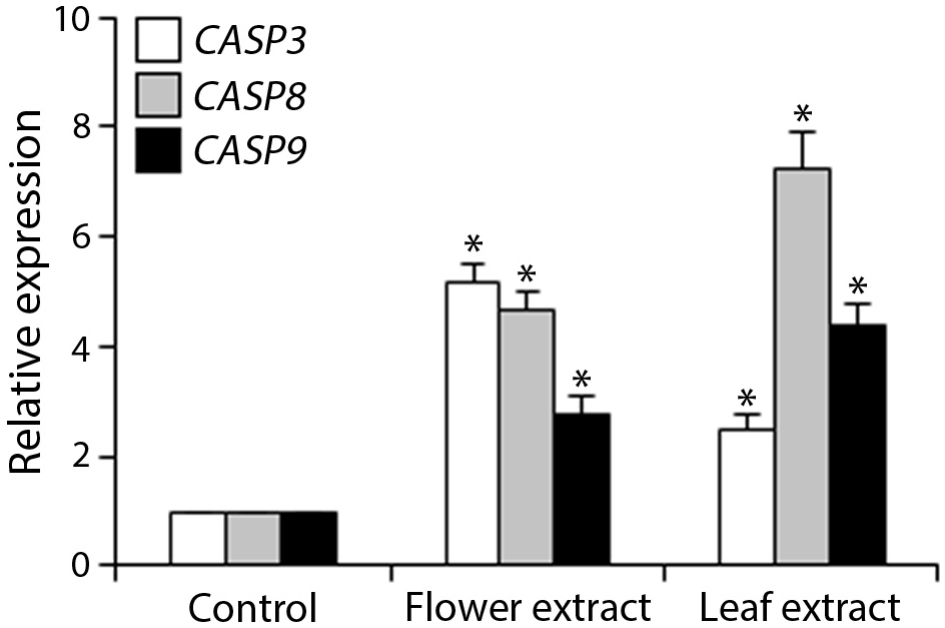
Gene expression levels of caspase genes in HeLa cells treated with IC50 of T. turcica extracts for 24 h. The asterisk (*) indicates significant differences as compared to control (P < 0.05).

The effect of FE and LE on the expression levels of caspase-8 ( *CASP8* ) and caspase-9 ( *CASP9* ) genes as well as the executioner caspase-3 ( *CASP3* ) is shown in Figure 3. The elevated transcript abundance of caspase genes was found in HeLa cells treated with IC50 of crude extracts. The expression level of *CASP8* was found higher as compared to *CASP9* . Additionally, a significant increase in the expression level of *CASP3* occurred in treated cells compared to untreated cells.

### 3.3. Proteomic analysis of HeLa cells exposed to T. turcica extracts

**Figure 4:**
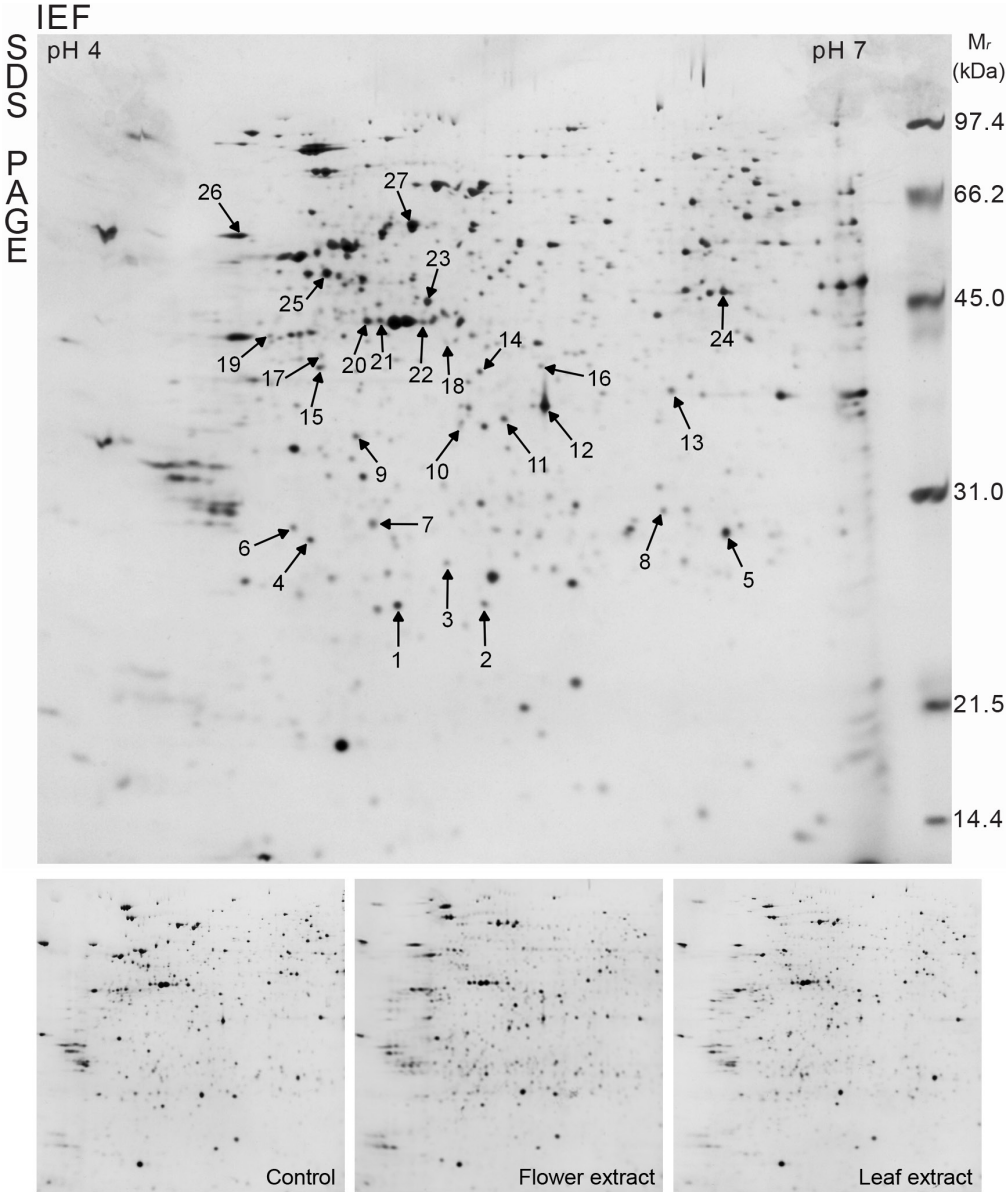
Representative 2-DE gel of differentially expressed protein in HeLa cells treated with flower and leaf extracts of T. turcica for 24 h. Total proteins were separated by 17 cm IPG strips (pH 4–7), following by 12% SDS-PAGE and silver staining.

**Figure 5:**
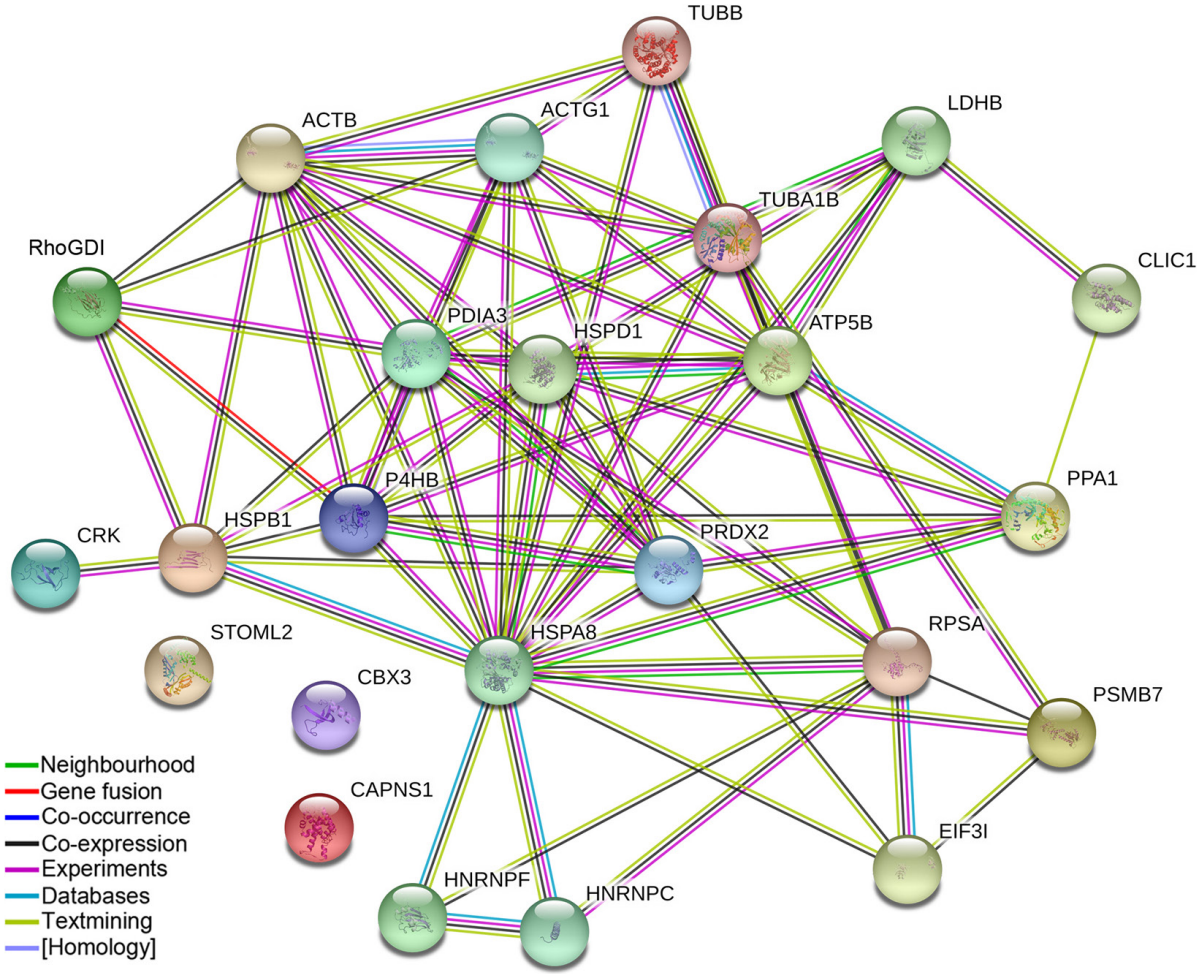
Protein interaction network for the differentially expressed proteins generated by STRING 9.1.

To better characterize the effects of *T. turcica* flower and leaf extracts on cervical carcinoma (HeLa) cells, proteomic analysis was used to identify specific proteins related to apoptosis. The alterations in proteomes of FE- and LE-treated HeLa cells were analyzed by 2-DE (Figure 4). Afterimage analyses, a total of 27 proteins were identified by MALDI-TOF/TOF MS analysis and MASCOT database searches (Table). The identified proteins have different biological functions including cytoskeleton organization (25.9%), protein metabolism (25.9%), chaperone activity (11.1%), energy metabolism (7.4%), and gene regulation (7.4%). Other categories are involved in chromatin regulator (3.7%), redox regulation (3.7%), apoptosis (3.7%), signal transduction (3.7%), primer metabolism (3.7%), and mitochondria metabolism (3.7%). Protein profiling revealed 16 up-regulated proteins and 11 down-regulated proteins in FE- and/or LE-treated HeLa cells. Two proteins, namely proteasome subunit beta type-7 (PSMB7, spot 8) and tubulin beta chain (TUBB, spot 16), were up-regulated in only LE-treated HeLa cells, while 4 proteins, including inorganic pyrophosphatase (PPA1, spot 11), suppressor of G2 allele of SKP1 homolog (spot 17), and actin 1 (ACTB, spots 20 and 21), were up-regulated in only FE-treated cells. Additionally, peroxiredoxin-2 (PRDX2, spot 2), adapter molecule crk (CRK, spot 3), chloride intracellular channel protein 1 (CLIC1, spot 9), tubulin beta chain (TUBB, spot 10), protein disulfide-isomerase A3 (PDIA3, spot 13), eukaryotic translation initiation factor 3 subunit I (EIF3I, spot 14), stomatin-like protein 2 (STOML2, spot 18), 40S ribosomal protein SA (RPSA, spot 19), actin 2 (ACTG1, spot 22), and protein disulfide isomerase (P4HB, spot 26) proteins were up-regulated in both FE- and LE-treated cells. However, Rho GDP-dissociation inhibitor 1 (RhoGDI, spot 4), heat shock protein beta-1 (HSBP1, spot 5), calpain small subunit 1 (CAPNS1, spot 3), heat shock cognate 71 kDa protein (HSPA8, spot 7), L-lactate dehydrogenase B chain (LDHB, spot 12), heterogeneous nuclear ribonucleoproteins C1/C2 (HNRNPC, spot 15) and F (HNRNPF, spot 23), tubulin alpha-1B chain (TUBA1B, spot 24), ATP synthase subunit beta mitochondrial (ATP5B, spot 25), and 60 kDa heat shock protein mitochondrial (HSPD1, spot 27) proteins were down-regulated in both FE- and LE-treated cells. One protein, namely chromobox protein homolog 3 (CBX3, spot 1), was down-regulated in only LE-treated cells. Furthermore, an interaction network of 27 differentially expressed proteins was constructed using STRING analysis (Figure 5).

**Table:** Table. Differentially expressed 27 proteins identified by MALDI-TOF/TOF mass spectrometry in HeLa cells exposed to leaf (LE) and flower (FE) extracts of T. turcica.

Spota	Accession numberb	Proteinc	Scored	MWpIe	Cover.f	MPg	Protein level h
1	CBX3_HUMAN	Chromobox protein homolog 3	316	20.85.23	46%	19	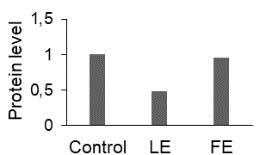
2	PRDX2_HUMAN	Peroxiredoxin-2	366	21.95.66	59%	22	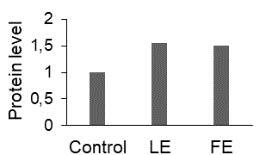
3	CRK_HUMAN	Adapter molecule crk	87	33.85.38	37%	15	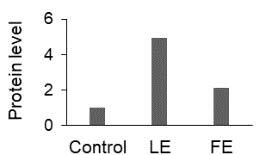
4	GDIR1_HUMAN	Rho GDP-dissociation inhibitor 1	532	23.25.02	47%	29	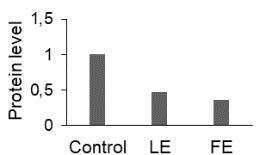
5	HSPB1_HUMAN	Heat shock protein beta-1	452	22.85.98	37%	27	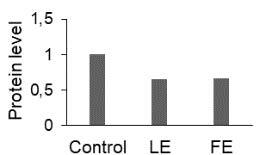
6	CPNS1_HUMAN	Calpain small subunit 1	189	28.35.05	35%	21	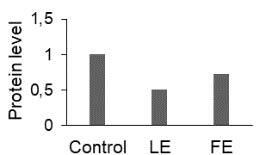
7	HSP7C_HUMAN	Heat shock cognate 71 kDa protein	543	70.95.37	33%	33	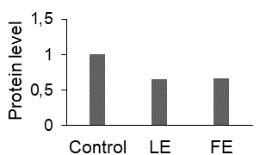
8	PSB7_HUMAN	Proteasome subunit beta type-7	92	29.97.57	21%	14	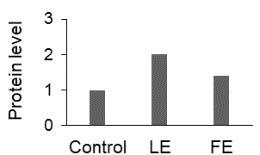
9	CLIC1_HUMAN	Chloride intracellular channel protein 1	527	26.95.09	63%	26	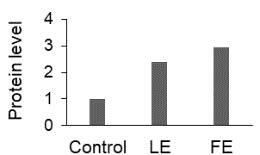
10	TBB5_HUMAN	Tubulin beta chain	385	49.64.78	38%	32	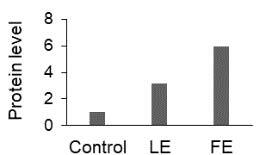
11	IPYR_HUMAN	Inorganic pyrophosphatase	389	32.65.53	54%	22	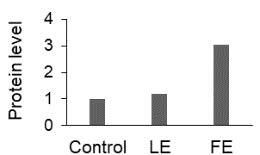
12	LDHB_HUMAN	L-lactate dehydrogenase B chain	277	36.65.71	33%	20	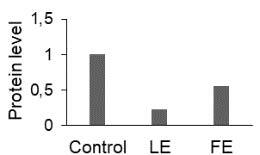
13	PDIA3_HUMAN	Protein disulfide-isomerase A3	686	56.75.98	54%	40	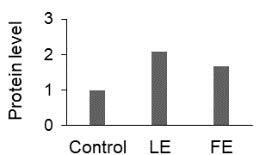
14	EIF3I_HUMAN	Eukaryotic translation initiation factor 3 subunit I	371	36.55.38	63%	28	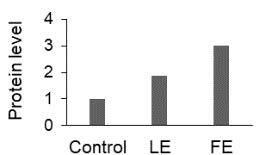
15	HNRPC_HUMAN	Heterogeneous nuclear ribonucleoproteins C1/C2	363	33.74.95	48%	26	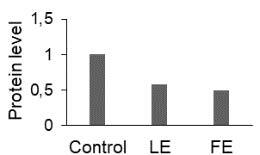
16	TBB5_HUMAN	Tubulin beta chain	280	49.64.78	39%	34	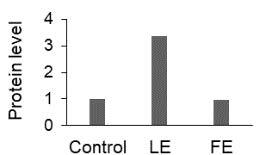
17	SUGT1_HUMAN	Suppressor of G2 allele of SKP1 homolog	155	40.95.07	35%	17	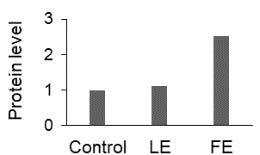
18	STML2_HUMAN	Stomatin-like protein 2	281	38.85.50	30%	33	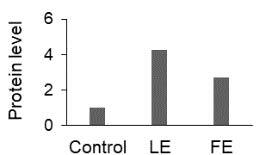
19	RSSA_HUMAN	40S ribosomal protein SA	478	32.84.79	58%	28	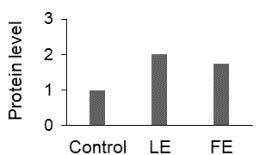
20	ACTB_HUMAN	Actin, cytoplasmic 1	468	41.75.29	56%	28	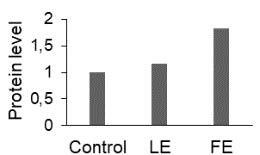
21	ACTB_HUMAN	Actin, cytoplasmic 1	270	41.75.29	45%	22	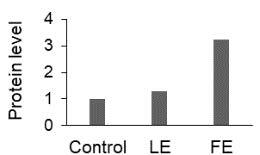
22	ACTG_HUMAN	Actin, cytoplasmic 2	447	41.85.31	56%	26	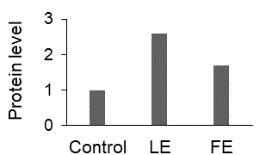
23	HNRPF_HUMAN	Heterogeneous nuclear ribonucleoprotein F	328	45.65.38	45%	28	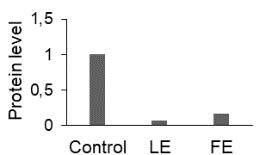
24	TBA1B_HUMAN	Tubulin alpha-1B chain	616	50.14.94	41%	27	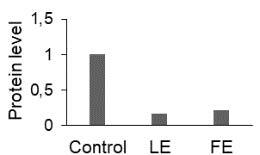
25	ATPB_HUMAN	ATP synthase subunit betaMitochondrial	779	56.55.26	53%	39	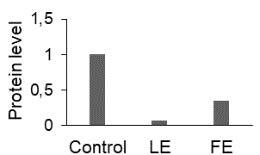
26	PDIA1_HUMAN	Protein disulfide-isomerase	369	57.14.76	41%	26	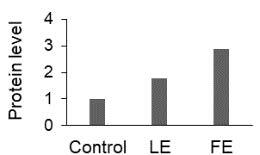
27	CH60_HUMAN	60 kDa heat shock proteinMitochondrial	665	61.05.70	41%	33	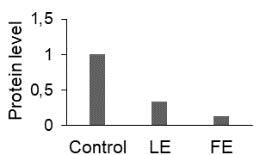

a Represents the spot number shown in 2-D gels (Figure 4).b Refers to the accession number according to the MASCOT search against the SwissProt database.c Refers to the protein name according to the MASCOT search against the SwissProt database.d Refers to the probability score for protein identification based on MS analysis and MASCOT search.e Refers to the molecular weight (MW, kDa) and isoelectric point (pI) for the protein identified.f Refers to the percentage of the matching amino acid sequence.g Refers to the number of matching peptide (MP).h Refers to changes in protein abundance.

## 4. Discussion

Natural products from plants with a broad range of biological activity potential can be a source of effective anticancer drugs [21]. Although a few studies reported the cytotoxicity of *T. turcica* extracts on some cancer types [11,12], there has been no research on the cytotoxic effects of different extracts in human cervical cancer. The results of the present study showed a significant inhibition of proliferation in *T. turcica* leaf and flower extracts, associated with induction of apoptosis as measured by DNA fragmentation and up-regulation of apoptotic genes. *T. turcica* is a rich source of natural products such as secondary metabolites [10]. Antitumor activities of plant-derived metabolites have been attributed to the plant secondary metabolites [4,22,23]. The present study suggests that these biologically active compounds of *T. turcica* could be responsible for its antiproliferative activity in HeLa cells.

Apoptosis is tightly regulated by extrinsic and intrinsic pathways. Caspase-8 is the initiator protease in the extrinsic pathway while caspase-9 is in the intrinsic pathway. These proteases promote the activity of the cascade of the effector caspases such as caspase-3 [24]. In the present study, crude extracts of *T. turcica* increased the gene expression of caspase-8, -9, and -3 in HeLa cells. Among the initiator caspases, the increase in the expression level of caspase-8 was more pronounced in an IC50 concentration. Caspase-3 is involved in the execution of the apoptotic pathway and activated by numerous death signals initiated by mitochondrial and nonmitochondrial pathways [25]. DNA fragmentation is largely mediated by the family of cysteinyl aspartate-specific protease caspases. The active form of caspase-3 protein occurs by post-translational cleavage [26]. Although the level of caspase-3 expression in the leaf extract is higher than the control, no apparent DNA fragmentation has been observed. This situation may be related to providing caspase-3 activity by the cleaved form of the protein. Additionally, proteomic analysis showed the down-regulation of Rho GDP-dissociation inhibitor 1 (RhoGDI) protein. RhoGDI is a major regulator of the activities of Rho GTPases and increases cancer cell survival under drug treatments by inhibiting the degradation of GTPase via caspases [27]. In the present study, reduced RhoGDI may induce apoptosis with the increased caspase-3 in HeLa cells treated with *T. turcica* extracts. Additionally, we found down-regulation of calpain small subunit 1 protein by proteomic analysis. Calpain is a calcium-dependent cysteine protease family, and it is implicated in the apoptotic pathway through interaction with caspases [28]. Atencio et al. [29] reported that inactivation of calpain in various cancer cells led to p53-dependent apoptosis, cell cycle arrest, and activation of caspase including caspase-8 and -9. It has been also shown that calpain inactivates caspase-9 by the degradation of enzymes [30]. Taken together, opposite expression of caspase genes and calpain in treated HeLa cells may be related to decreased cell proliferation.

Chromobox protein homolog 3 (CBX3) has been involved in the regulation of gene transcription, and it is upregulated in many cancer types [31,32]. It is also suggested that CBX3 plays a crucial role in the progression of tumors [33]. CBX3 promotes cell proliferation in colon cancer and tongue squamous cell carcinoma by suppressing the p21 activity [31,34]. It is also stated that CBX3 mediates the recruitment of NIPBL (Nipped-B-like protein) to sites of DNA damage [35]. In our study, the abundance of CBX3 protein was down-regulated in LE-treated HeLa cells. This result suggests that leaf extract harms DNA repair mechanisms and cell proliferation in HeLa cells by suppressing the CBX3 protein.

Heat shock proteins (HSPs) function as chaperones that help the abnormal proteins formed under stress conditions to fold properly [36]. HSPs are essential for cell metabolism and are considered as the protector of oncoprotein related to cancer proliferation and progression [37]. HSPs function as antiapoptotic proteins and high levels of HSPs have been associated with aggressiveness of several tumors [38,39]. The down-regulation of three HSP proteins (HSBP1, HSPA8, and HSPD1) may associate with a cytotoxic response of *T. turcica* extracts in HeLa cells.

Protein disulfide isomerase (PDI) family proteins catalyze the formation of disulfide bonds [40]. ER stress often results in increased regulation of chaperones, such as the PDI family, which serves as an important cellular defense against abnormal proteins [41]. Liu et al. [42] reported that the constant ER stress triggers the signaling switch from prosurvival to proapoptosis by the release of PDIs (PDIA1 and PDIA3) from the ER lumen to induce Bak-dependent mitochondrial outer membrane permeabilization. In our study, leaf and flower extracts of *T. turcica* up-regulated the expression levels of PDI proteins (PDIA1 and PDIA3) as well as proteasome subunit beta type-7. This result shows that *T. turcica* extracts can induce the Bak-dependent apoptotic pathway by releasing the PDIs from the ER lumen to the cytosol.

Heterogeneous nuclear ribonucleoprotein (HNRNP) has been reported to promote the expression of c-Myc and Bag-1 proteins [43]. Down-regulation of c-Myc is associated with the stimulation of apoptosis in some cancer cells [44,45]. It is also reported that suppression of c-Myc expression led to G2/M arrest in different cell types [46, 47]. In our study, the lower abundance of HNRNP (HNRNPC and HNRNPF) proteins may be responsible for the cell cycle inhibiting and apoptotic effects of *T. turcica* extracts by inhibiting the expression of c-Myc.

Stomatin-like protein 2 (STOML2) is a major component of the inner membrane of mitochondria [48]. Previous studies revealed the prognostic role of STOML2 protein in different cancer types including cervical cancer [49,50]. Suppression of STOML2 in esophageal cancer cells reduced the growth rate and cell attachment [51]. In the present study, the expression level of STOML2 protein was up-regulated in HeLa cells treated with *T. turcica* extracts. However, further studies are necessary for the determination of the mechanism of STOML2 protein in HeLa cells.

Under hypoxia, the pyruvate is converted to lactate in cancer cells by lactate dehydrogenase (LDHB) [52]. The expression of LDHB was significantly increased in some kinds of tumors [53,54]. The down-regulation of LDHB protein in *T. turcica* treated HeLa cells may suggest inhibition of cell proliferation. Since NAD+ is required for glycolysis, inhibition of LDHB could lead to cellular energy collapse and subsequent cell death.

In conclusion, the methanolic extracts of leaves and flowers of *T. turcica* cause cytotoxic effects in HeLa cancer cell lines. Gene expression levels of caspase-3, -8, and -9 proteases which play a role in apoptosis were significantly increased in HeLa cell lines treated with *T. turcica* extracts. This is the first comparative proteomic research providing molecular insights about the bioactivity of *T. turcica* extracts in HeLa cells. The expression levels of 27 proteins were regulated differently in HeLa cells treated with *T. turcica* extracts. It can be suggested that different levels of proteins such as HSPs, PDI, RhoGDI, CBX3, and HNRNP can contribute to cell cycle inhibiting and apoptotic effects of *T. turcica* extracts. The results of this research have provided valuable proteomic information about the anticancer activity of *T. turcica* extracts in human cervical cancer.

## References

[ref1] (2015). Cancer incidence and mortality worldwide: sources, methods and major patterns in GLOBOCAN 2012. International Journal of Cancer.

[ref2] (2011). Human papillomavirus testing in the prevention of cervical cancer. Journal of the National Cancer Institute.

[ref3] (2017). Cervical cancer: A global health crisis. Cancer.

[ref4] (2018). Plants as sources of natural and recombinant anti-cancer agents. Biotechnology Advances.

[ref5] (2015). Natural products potential and scope for modern cancer research. American Journal of Plant Sciences.

[ref6] (2013). Anticancer peptides and proteins: a panoramic view. Protein and Peptide Letters.

[ref7] (2003). A toxicity experiment of general alkaloid of Thermopsis lanceolate on mice. Journal of Northwest Sci-Tech University of Agriculture and Forestry.

[ref8] (2015). Natural product extracts of the Canadian prairie plant, Thermopsis rhombifolia, have anti-cancer activity in phenotypic cell-based assays. Natural Product Research.

[ref9] (2012). Determination of mutagenic potencies of aqueous extracts of Thermopsis turcica by Ames test. Turkish Journal of Biology.

[ref10] (2013). Free radical scavenging activity, total phenolic content, total antioxidant status and total oxidant status of endemic Thermopsis turcica. Saudi Journal of Biological Sciences.

[ref11] (2014). Antimicrobial activity against periodontopathogenic bacteria, antioxidant and cytotoxic effects of various extracts from endemic Thermopsis turcica. Asian Pacific Journal of Tropical Biomedicine.

[ref12] (2017). Anticancerous efficacy of alcoholic and aqueous extracts from an endemic plant Thermopsis turcica on liver carcinoma. Journal of Pharmaceutical Research International.

[ref13] (2014). Proteomic investigation into betulinic acid-induced apoptosis of human cervical cancer HeLa cells. PLoS ONE.

[ref14] (2016). In situ proteomic profiling of curcumin targets in HCT116 colon cancer cell line. Scientific Reports.

[ref15] (2018). Proteomic analysis reveals that an extract of the plant Lippia origanoides suppresses mitochondrial metabolism in triple-negative breast cancer cells. Journal of Proteome Research.

[ref16] (2017). Flower extract of Allium atroviolaceum triggered apoptosis, activated caspase-3 and down-regulated antiapoptotic Bcl-2 gene in HeLa cancer cell line. Biomedicine and Pharmacotherapy.

[ref17] (1976). A rapid and sensitive method for the quantification of microgram quantities of protein utilizing the principle of protein-dye binding. Analytical Biochemistry.

[ref18] (2001). A new silver staining apparatus and procedure for matrix-assisted laser desorption/ionization-time of flight analysis of proteins after two-dimensional electrophoresis. Proteomics.

[ref19] (2004). Blue silver: a very sensitive colloidal Coomassie G-250 staining for proteome analysis. Electrophoresis.

[ref20] (2011). The STRING database in 2011: functional interaction networks of proteins, globally integrated and scored. Nucleic Acids Research.

[ref21] (2018). Plant secondary metabolites as anticancer agents: Successes in clinical trials and therapeutic application. International Journal of Molecular Sciences.

[ref22] (2014). Plant-derived epigenetic modulators for cancer treatment and prevention. Biotechnology Advances.

[ref23] (2016). Antiproliferative and computational studies of two new pregnane glycosides from Desmidorchis flava. Bioorganic Chemistry.

[ref24] (2013). Programmed cell death: molecular mechanisms and implications for safety assessment of nanomaterials. Accounts of Chemical Research.

[ref25] (2003). Caspase 3 in breast cancer. Clinical Cancer Research.

[ref26] (2010). Regulation of DNA fragmentation: the role of caspases and phosphorylation. FEBS Journal.

[ref27] (2005). dissociation inhibitor protects cancer cells against drug-induced apoptosis. Cancer Research.

[ref28] (2003). The calpain system. Physiological Reviews.

[ref29] (2000). Calpain inhibitor 1 activates p53-dependent apoptosis in tumor cell lines. Cell Growth and Differentiation.

[ref30] (2000). Direct cleavage by the calcium- activated protease calpain can lead to inactivation of caspases. Journal of Biological Chemistry.

[ref31] (2017). CBX3 promotes colon cancer cell proliferation by CDK6 kinase-independent function during cell cycle. Oncotarget.

[ref32] (2018). CBX3 promotes proliferation and regulates glycolysis via suppressing FBP1 in pancreatic cancer. Biochemical and Biophysical Research Communications.

[ref33] (2019). CBX3 predicts an unfavorable prognosis and promotes tumorigenesis in osteosarcoma. Molecular Medicine Reports.

[ref34] (2018). CBX3 promotes tumor proliferation by regulating G1/S phase via p21 downregulation and associates with poor prognosis in tongue squamous cell carcinoma. Gene.

[ref35] (2017). Independent mechanisms recruit the cohesin loader protein NIPBL to sites of DNA damage. Journal of Cell Science.

[ref36] (2000). Heat shock proteins—modulators of apoptosis in tumour cells. Leukemia.

[ref37] (2019). Proteomic analysis of anticancer effects of Streblus asper root extract on HeLa cancer cells. Biomedical and Pharmacology Journal.

[ref38] (2012). Malinowsky. PLoS ONE.

[ref39] (2012). Targeting heat shock protein 27 (HspB1) interferes with bone metastasis and tumor formation in vivo. British Journal of Cancer.

[ref40] (2005). The human protein disulphide isomerase family: substrate interactions and functional properties. EMBO Reports.

[ref41] (2014). Mollereau B. Nature Reviews Neuroscience.

[ref42] (2017). Effect of subcellular translocation of protein disulfide isomerase on tetrachlorobenzoquinone-induced signaling shift from endoplasmic reticulum stress to apoptosis. Chemical Research in Toxicology.

[ref43] (2006). The roles of heterogeneous nuclear ribonucleoproteins in tumour development and progression. Biochimica et Biophysica Acta.

[ref44] (1998). The many roles of c-Myc in apoptosis. Annual Review of Physiology.

[ref45] (2008). Suppression of tumorigenesis by human mesenchymal stem cells in a hepatoma model. Cell Research.

[ref46] (2005). Iron specific growth inhibition of Burkitt’s lymphoma cells in vitro, associated with a decrease in translocated c-myc expression. Journal of Cellular Physiology.

[ref47] (2006). Structural insight of human DEAD-box protein rck/p54 into its substrate recognition with conformational changes. Genes to Cells.

[ref48] (2000). Identification and characterization of human slp-2, a novel homologue of stomatin (band 7.2b) present in erythrocytes and other tissues. Journal of Biological Chemistry.

[ref49] (2015). Stomatin-like protein 2 is overexpressed in epithelial ovarian cancer and predicts poor patient survival. BMC Cancer.

[ref50] (2015). Stomatin-like protein 2 expression is associated with clinical survival in patients with cervical cancer. International Journal of Clinical and Experimental Pathology.

[ref51] (2006). Stomatin-like protein 2 is overexpressed in cancer and involved in regulating cell growth and cell adhesion in human esophageal squamous cell carcinoma. Clinical Cancer Research.

[ref52] (2008). Tumor metabolism: cancer cells give and take lactate. Journal of Clinical Investigation.

[ref53] (2011). Mitochondrial and plasma membrane lactate transporter and lactate dehydrogenase isoform expression in breast cancer cell lines. Physiological Genomics.

[ref54] (2012). Importance of glycolysis and oxidative phosphorylation in advanced melanoma. Molecular Cancer.

